# Circularization of flavivirus genomic RNA inhibits *de novo* translation initiation

**DOI:** 10.1093/nar/gkz686

**Published:** 2019-08-08

**Authors:** Thomas J Sanford, Harriet V Mears, Teodoro Fajardo, Nicolas Locker, Trevor R Sweeney

**Affiliations:** 1 Division of Virology, Department of Pathology, University of Cambridge, Addenbrooke's Hospital, Hills Road, Cambridge, CB2 0QQ, UK; 2 Faculty of Health and Medical Sciences, School of Biosciences and Medicine, University of Surrey, Guildford, GU2 7HX, UK

## Abstract

Members of the *Flaviviridae* family, including dengue virus (DENV) and yellow fever virus, cause serious disease in humans, whilst maternal infection with Zika virus (ZIKV) can induce microcephaly in newborns. Following infection, flaviviral RNA genomes are translated to produce the viral replication machinery but must then serve as a template for the transcription of new genomes. However, the ribosome and viral polymerase proceed in opposite directions along the RNA, risking collisions and abortive replication. Whilst generally linear, flavivirus genomes can adopt a circular conformation facilitated by long-range RNA–RNA interactions, shown to be essential for replication. Using an *in vitro* reconstitution approach, we demonstrate that circularization inhibits *de novo* translation initiation on ZIKV and DENV RNA, whilst the linear conformation is translation-competent. Our results provide a mechanism to clear the viral RNA of ribosomes in order to promote efficient replication and, therefore, define opposing roles for linear and circular conformations of the flavivirus genome.

## INTRODUCTION

Zika virus (ZIKV) is a mosquito-borne flavivirus which causes severe congenital birth defects, including neonatal microcephaly, and has been associated with autoimmune Guillain–Barré syndrome (1,2). Other members of the *Flavivirus* genus, including dengue virus (DENV), West Nile virus (WNV) and yellow fever virus pose major risks to public health, particularly in South-East Asia, Africa and Latin America (3,4). Coupled with the expanding habitat ranges of their insect vectors, the pandemic re-emergence of these pathogens makes them a continuing matter of global concern (5,6).

Flaviviruses have a single-stranded positive-sense RNA genome, which serves as both the message for translation and template for replication (7,8). As such, the same RNA is demanded by both the ribosome and viral RNA-dependent RNA polymerase (NS5^pol^), which inherently progress in opposite directions, presenting the likelihood of a collision and abortive translation or replication products. This has led to the model of a ‘lifestyle switch’ whereby, following initial rounds of viral protein synthesis, translation of the viral RNA genome is inhibited freeing the RNA of ribosomes so that it can efficiently serve as a template for negative strand synthesis. Indeed, it has been demonstrated for poliovirus, a single-stranded positive-sense RNA picornavirus, that translation and replication are mutually exclusive, with cleavage of trans-acting factors required for translation initiation by a virally encoded protease serving as a switch to promote temporal viral genome usage (9–11). Whilst it has been shown for DENV that translation and replication both occur in association with the ER, the trigger to switch between these two processes is undefined (12).

Members of other genera within the *Flaviviridae* family, most notably hepatitis C virus (HCV) and classical swine fever virus (CSFV), contain an internal ribosome entry site (IRES) to facilitate translation initiation (13). Instead, owing to the presence of a virally encoded methylguanosine cap at the 5′ end of the flavivirus genome, it has been assumed that flaviviral translation during infection occurs in a predominantly canonical cap-dependent manner (8). In eukaryotes, this starts with the formation of a 43S pre-initiation complex, comprising the 40S ribosomal subunit, eukaryotic translation initiation factor (eIF)3, ternary complex (eIF2/GTP/Met-tRNA_i_^Met^), eIF1 and eIF1A (14). Recruitment of the 43S pre-initiation complex to the mRNA is facilitated by the 5′ cap-binding complex eIF4F. The helicase eIF4A is a component of eIF4F and, stimulated by its cofactor eIF4B, is responsible for unwinding RNA secondary structure in the 5′ untranslated region (UTR) (15). Highly structured 5′ UTRs may require additional helicases, such as the DExH-box protein DHX29, which is essential for efficient initiation on Aichi virus and Sindbis virus mRNAs (16,17). Once unwound, the 5′ UTR can then be scanned until the 43S complex recognizes a suitable AUG start codon in good nucleotide context (18), the fidelity of which is regulated by eIF1 and eIF1A (19). Following start codon recognition and partial hydrolysis of eIF2-bound GTP, a stable 48S complex is formed. eIF5B subsequently promotes dissociation of initiation factors and recruitment of the 60S ribosomal subunit ready for polypeptide elongation (20).

**Figure 1.**
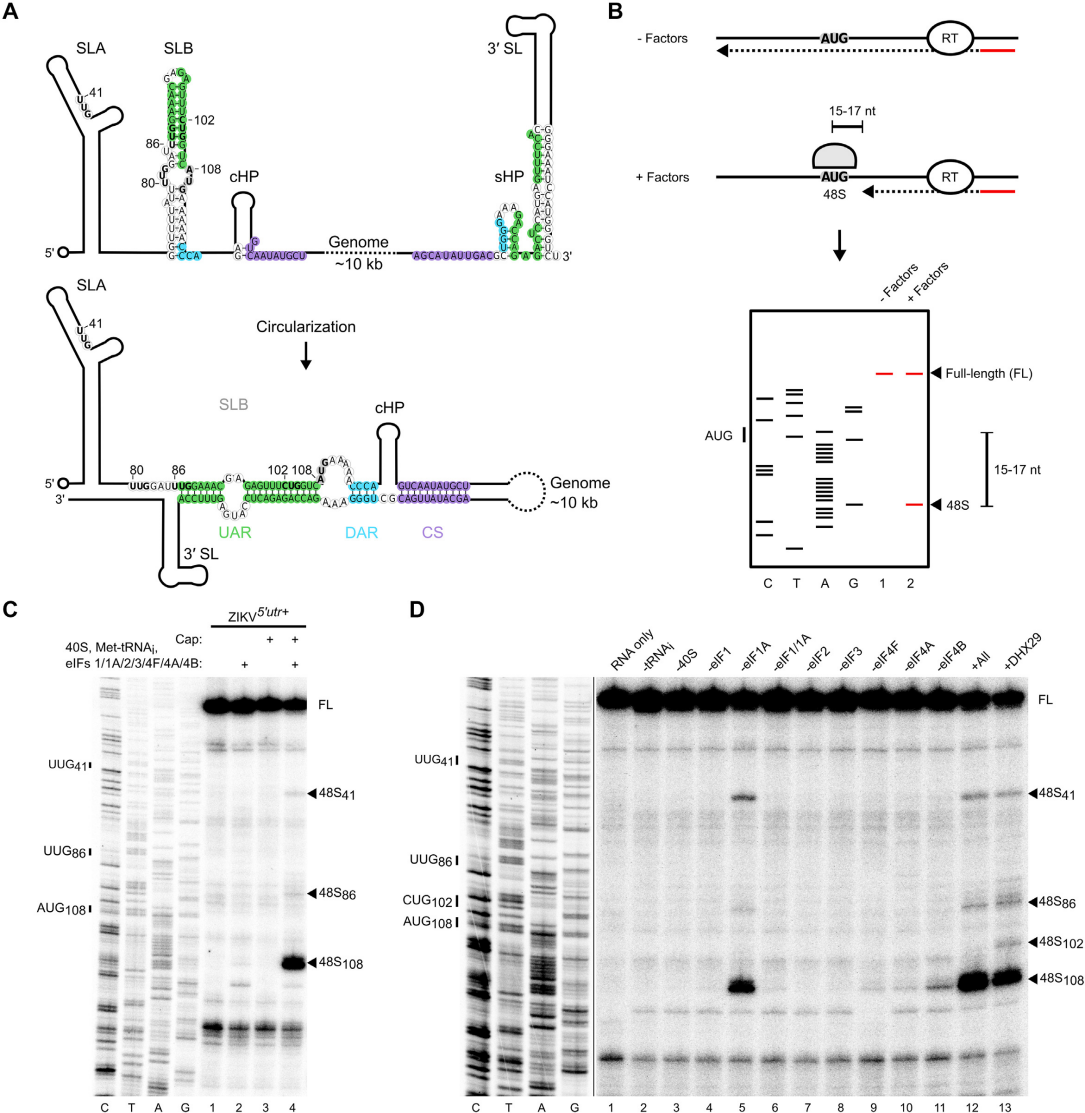
48S complex assembly on ZIKV RNA is cap-dependent. (**A**) Schematic of the ZIKV RNA in a linear (upper) and circular (lower) conformation. The nucleotides corresponding to the UAR, DAR and CS are shaded in green, blue and purple, respectively. The initiating AUG and upstream near-cognate codons discussed in the text are in bold. (**B**) Schematic representation of the *in vitro* reconstitution and toeprinting assay described in the text. (**C** and **D**) Toeprinting analysis of 48S complex assembly on (C) uncapped or capped ZIKV*^5′utr+^* RNA and (D) capped ZIKV*^5′utr+^* RNA in the absence of the indicated factors. Selected codons are labelled on the left and toeprints caused by 48S complex assembly are marked with a closed arrowhead on the right. To balance the intensities in panel D, different exposures of the sequencing and toeprint portions of the gel are shown, indicated by the black line. FL, full-length. See also Supplementary Figure S1.

The 5′ UTRs of flaviviral genomes are highly structured, with three major conserved regions: stem loop A (SLA), stem loop B (SLB) and a short hairpin in the capsid-coding region (cHP) (21) (Figure 1A). Particular DENV serotypes, whose start codons are in poor nucleotide context, require the cHP to facilitate start codon selection by arresting the scanning ribosome over the correct AUG, which is typically located within SLB (22). Flaviviruses do not possess a 3′-polyA tail, but instead have a highly structured 3′ UTR (Figure 1A) which in DENV can recruit the polyA-binding protein (23). This was hypothesized to promote genomic RNA circularization and encourage translation re-initiation in a manner similar to the ‘closed-loop’ model proposed for cellular mRNAs (24). However, flavivirus genomes can also circularize by means of long-range RNA–RNA interactions between elements at the 5′ and 3′ ends (Figure 1A). These are comprised of the upstream of AUG region (UAR), the downstream of AUG region (DAR) and the cyclization sequence (CS) (25–28). Since the 5′ UAR element forms the majority of SLB within the linear conformation, genome circularization involves dissolution of this structure in favour of duplex formation with the 3′ end. This process is essential for viral genome replication, since the viral NS5^pol^ is recruited to SLA in the 5′ UTR before translocating to the 3′ end to initiate negative strand synthesis facilitated by the close proximity of the two genomic ends in the circularized form (29,30). Consistently, it was reported that mutations which stabilize SLB in DENV inhibit both genome circularization and replication (31).

Recent in-cell RNA structure mapping has demonstrated that the ZIKV genome predominantly adopts a linear conformation during infection (32). Given that circular RNA is more efficiently replicated, a role for this linear form is unclear. However, mutations in the UAR of DENV that stabilize the circular over the linear form actually inhibit viral replication (33), implying that linear RNAs are required at different stages of the viral life cycle. It is possible the transition between linear and circular conformations is a critical regulatory step during initiation of replication, since *in vitro* NS5^pol^ is more efficiently recruited to the linear form of the viral RNA (31). However, it is still unknown how this major 5′ UTR remodelling, particularly in regions flanking the initiating AUG, affects viral translation.

Here, we present a detailed mechanistic analysis of the requirements for translation initiation on a cap-dependent flavivirus. Using an *in vitro* reconstitution approach, we demonstrate that the linear conformation of the flavivirus genomic RNA is highly translatable, but genome circularization inhibits *de novo* translation initiation on ZIKV and DENV RNAs. Decreased initiation at the viral main open reading frame start codon occurs as a result of defective ribosomal scanning, induced by the RNA structure formed upon hybridization of the 5′ and 3′ ends of the genomic RNA. This provides a mechanism to promote clearance of the ribosome from replication-competent RNAs. Therefore, we propose a model in which genome circularization acts as a barrier to prevent translation during genome replication to maintain an obstruction-free template for RNA synthesis.

## MATERIALS AND METHODS

### Plasmids and reagents

Recombinant human eIF expression plasmids have been described previously: eIF1 and eIF1A (19), eIF4A and eIF4B (34), eIF4G_736–1115_ (35), DHX29 (36) and methionyl-tRNA synthetase (37). The eIF4E expression plasmid was a kind gift from Stephen Curry. The tRNA_i_^Met^ transcription vector has been described (38). The classical swine fever (CSFV) IRES plasmid was previously described (39). A pCC1BAC vector containing the open reading frame of the ZIKV BeH819015 isolate flanked by the 5′ and 3′ UTRs of the ZIKV PE243 isolate and a related plasmid containing an inline duplicate copy of the capsid protein fused to a Nluc gene and 2A peptide sequence (40) were kindly provided by Andres Merits. The existing SP6 promotor in these plasmids was replaced with a T7 promotor by subcloning a synthetic gene fragment (Integrated DNA Technologies; IDT) between EcoRI and NheI sites. NS5^pol^ G_664_AA and Δ3′ CS mutations were generated by overlap extension polymerase chain reaction (PCR) to produce a fragment that was inserted between BstBI and PmeI sites. pUC57-ZIKV*^mini^* was derived from pUC57-ZIKV-Fluc (41) by site-directed mutagenesis; 30 nt from the 5′ and 3′ ends of the Fluc gene were retained. Mutations in ZIKV*^mini^* were also generated by site-directed mutagenesis. Gene fragments containing corresponding wild-type (WT) and Δ3′ CS mutant DENV1 (KC692517.1) or DENV4 (FJ196850.1) minigenomes, or the first 359 nt of ZIKV PE243 (KX197192.1), with flanking XbaI and EcoRV sites (DENV1/4) or XbaI and HindIII sites (ZIKV^*5′utr+*^) and 5′ T7 promotor, were synthesized by IDT and cloned into pUC57. Rocaglamide was purchased from Sigma and hippuristanol was generously shared by Jerry Pelletier.

### *In vitro* transcription

Plasmids were linearized with HindIII (ZIKV*^5′utr+^*and ZIKV*^mini^*), EcoRV (DENV1/4*^mini^*), AgeI (ZIKV^*Nluc*^, ZIKV*^fl^*), EcoRI (CSFV) or BstN1 (tRNA_i_^Met^). RNAs were transcribed with recombinant T7 polymerase (50 ng/μl) in buffer containing 40 mM HEPES pH 7.5, 32 mM MgOAc, 40 mM dithiothreitol (DTT), 2 mM Spermidine, 7.5 mM each nucleotide triphosphate (NTP) and 0.2 U/μl RNaseOUT (Invitrogen) for 2 h at 37°C. Scrambled and targeted antisense RNAs were similarly transcribed from T7 promoter-bearing reverse complement DNA oligos annealed to a T7 promoter-containing forward primer. Sequences are shown in Supplementary Table S1. ZIKV*^fl^* and ZIKV*^Nluc^* RNA were DNaseI treated and purified using TRI Reagent (Sigma) before ethanol precipitation. All other transcription reactions were treated with DNaseI and RNA was extracted with acidic phenol/chloroform and ethanol precipitated. Residual nucleotides were removed with Illustra MicroSpin G-50 columns (GE Healthcare). RNA was capped using the ScriptCap system (CellScript).

### Purification of initiation factors and aminoacylation of tRNA

40S ribosomal subunits, eIF2, eIF3 and eIF4F were purified from rabbit reticulocyte lysate (RRL) as previously described (42). Recombinant eIF1, eIF1A, eIF4A, eIF4B, eIF4G_736–1115_, eIF4E and methionyl-tRNA synthetase were expressed in *Escherichia coli* BL21 (DE3) Star and purified by affinity chromatography on Ni-NTA agarose beads (Qiagen) and polished by FPLC on monoQ or monoS (GE healthcare), as previously described (42,43). DHX29 was purified by affinity chromatography on Ni-NTA agarose beads followed by anti-FLAG M2 affinity gel (Sigma) and eluted with FLAG peptide (Sigma), as described (36). Recombinant tRNA_i_^Met^ was charged with methionine, as previously described (42).

### Assembly and analysis of ribosomal complexes

48S complexes were assembled as previously described (44). 0.2 pmol RNA was incubated with the indicated eIFs (2 pmol 40S subunit, 4 pmol Met-tRNA_i_^Met^, 4 pmol eIF2, 3 pmol eIF3, 10 pmol eIF4A, 5 pmol eIF4B, 5 pmol eIF4G_736-1115_, 2.5 pmol eIF4F, 10 pmol eIF1, 10 pmol eIF1A, 0.4 pmol DHX29) at 37°C for 10 min in a reaction volume of 20 μl containing 20 mM Tris pH 7.5, 100 mM KCl, 2.5 mM MgCl_2_, 2 mM DTT, 0.25 mM spermidine, 1.6 U/μl RNaseOUT (Invitrogen), 0.4 mM guanosine triphosphate (GTP) and 2 mM adenosine triphosphate. Assembled complexes were analysed by primer extension inhibition using 2.5 U avian myeloblastosis virus reverse transcriptase (AMV-RT) (Promega) in the presence of ^32^P-labelled primer, 8 mM MgCl_2_ and 0.5 mM dNTPs. cDNA products were phenol/chloroform extracted and ethanol precipitated before being resolved on denaturing 6% polyacrylamide sequencing gels and detected by autoradiography using an FLA7000 Typhoon scanner (GE).

### RNA EMSA

*In vitro* transcribed WT and mutant minigenome RNAs (0.5 pmol) were subjected to 4.5% native polyacrylamide gel electrophoresis (PAGE) at 100 V for 2.5 h at 4°C. Antisense or scrambled RNA oligos (2.4 pmol) were annealed to minigenome RNAs (1.2 pmol) by heating to 75°C in the presence of a RNA refolding buffer (50 mM Tris pH 7.5, 100 mM KCl and 5 mM MgCl_2_), then snap cooled on ice. Gels were stained with 1 μg/ml ethidium bromide and visualized using a UV transilluminator.

### SHAPE reactivity reactions

RNA (50 nM) was incubated in the presence or absence of 10 mM *N*-methylisatoic anhydride (NMIA) (ThermoFisher) in 10 μl of buffer containing 30 mM Tris pH 7.5, 100 mM KCl and 2.5 mM MgCl_2_ for 45 min at 37°C. RNA was subsequently phenol/chloroform extracted and ethanol precipitated. Modified bases were detected by inhibition of primer extension using 2.5 U AMV-RT in the presence of ^32^P-labelled primer and 0.5 mM dNTPs. cDNA products were phenol/chloroform extracted and ethanol precipitated before being resolved on denaturing 6% polyacrylamide sequencing gels and detected by autoradiography using an FLA7000 Typhoon scanner (GE).

### SHAPE data analysis

Resolved cDNA products were quantified using ImageQuant TL software (GE). Band intensities from sample lanes not treated with NMIA were subtracted from those treated with NMIA to obtain selective 2′-hydroxyl acylation analysed by primer extension (SHAPE) reactivity. For each experiment the top 2% of SHAPE reactivities obtained for nucleotides 117–160, which were quantifiable for all RNAs examined, were excluded and the top 10% of the remaining SHAPE reactivities averaged. Reactivities at other positions were then normalized to this average with 0 representing unreactive nucleotides and 1 as highly reactive. Data from three experiments was analysed and standard deviations calculated in GraphPad Prism v7. Statistical significance of differences between the SHAPE reactivities of nucleotides corresponding to the 5′ CS sequence was determined from three independent experiments using the unpaired two-tailed Student's *t*-test as calculated in GraphPad Prism v7 and displayed in Supplementary Table S2.

### RNA transfection

Capped (cap0), *in vitro* transcribed ZIKV*^Nluc^* RNA (5 μg) was electroporated into 3 × 10^6^ Vero cells suspended in 100 μl of Opti-MEM (Gibco) using a NEPA21 electroporator (Nepagene). Cells were seeded sub-confluently in 24-well plates and samples were harvested for luciferase assays and RT-qPCR analysis at the indicated time points by washing with phosphate-buffered saline and lysis in passive lysis buffer (Promega).

### Luciferase assay

Nanoluciferase activity was measured using the Nano-Glo luciferase assay system (Promega) by GloMax (Promega). Luciferase signal was normalized to protein concentration, determined by bicinchoninic acid (BCA) protein assay kit (Pierce).

### RNA extraction and RT-qPCR

Total RNA was extracted from cell lysates by adding a 2:1 ratio of TRI Reagent (Sigma). Subsequent extraction was achieved following the manufacturer's instructions, except isopropanol precipitation was performed in the presence of 25 ng/μl GlycoBlue (Invitrogen) and the resulting pellet was washed twice with 75% ice cold ethanol to remove contaminating phenol and salts. Pellets were resuspended in water and RNA was quantified by measuring absorbance at 260 nm using a NP80 spectrometer (Implen). The number of viral genome copies in each sample was quantified by RT-qPCR analysis using TaqMan chemistry with a Genesig Standard Zika virus quantification kit (Primer Design) and PrecisionPLUS OneStep 2× RT-qPCR reagent (Primer Design) on a StepOnePlus real-time PCR system (Applied Biosystems). Per reaction, 0.3 μl primer/probe mix, 5 μl of 2× RT-qPCR reagent and 4.7 μl sample were used ensuring that the total amount of RNA in each reaction was <500 ng. Cycling parameters consisted of an RT step (50°C, 30 min), an initial denaturation step (95°C, 5 min) and then 45 cycles of amplification (95°C, 15 s; 60°C extension, 1 min). Ct values for each sample were calculated using fluorescence data and compared to a standard curve derived from dilutions of known ZIKV genome copy number generated for each plate analysed. Amplification of no template controls was not observed.

## RESULTS

### *In vitro* reconstitution of Zika virus translation initiation

To investigate the mechanism of flavivirus translation, we reconstituted translation initiation on ZIKV RNA *in vitro*. This approach has previously been used to successfully probe the mechanism of cellular cap-dependent and viral IRES-dependent translation (44). EIFs and small ribosomal subunits were individually purified, either natively from RRL or recombinantly from bacteria. EIFs were incubated with an *in vitro* transcribed RNA bearing the ZIKV 5′ UTR and first 252 nt of the capsid protein (ZIKV*^5′utr+^*). 48S complex assembly was assayed by primer extension inhibition, since assembled complexes arrest reverse transcriptase resulting in a truncated cDNA product 15–17 nt downstream of the initiating codon, as identified by concomitant Sanger sequencing (42). A schematic of this technique is shown in Figure 1B.

We investigated the impact of a 5′ cap structure on the efficiency of translation initiation on ZIKV RNA in the reconstituted system. In the presence of the full complement of canonical translation initiation factors, very weak 48S complex formation is detected when ZIKV*^5′utr+^* RNA is uncapped (Figure 1C, lane 2). Capping of the RNA results in 48S complex formation at the authentic AUG_108_ as determined by the increase in the toeprint signal (Figure 1C, compare lanes 2 and 4).

We next sought to determine which eIFs are necessary for ZIKV translation initiation by performing systematic factor omission on capped ZIKV^5′^*^utr+^* RNA. Omission of eIF2 and eIF3 completely inhibited translation initiation at AUG_108_ (Figure 1D, lanes 7 and 8), whilst very weak 48S complex formation occurred in the absence of eIF4F (Figure 1D, lane 9), consistent with the requirement for a 5′ cap for translation. EIF4E, in the presence or absence of a fragment of eIF4G containing residues 736–1115 (eIF4G_736–1115_) that supports translation initiation on Type 1 and Type 2 picornaviral IRESs (44), could not compensate for eIF4F (Supplementary Figure S1A). In comparison, using the same set of purified factors, 48S complex assembly on the HCV-like IRES from CSFV was detected when only small ribosomal subunits, eIF2 and Met-tRNA_i_^Met^ (Supplementary Figure S1B) were included as previously described (39), confirming that these factors are sufficient for initiation on IRESs of viruses from other genera in the *Flaviviridae*. ZIKV translation initiation was similarly abrogated by the absence of eIF1 (Figure 1D, lanes 4 and 6). However, we observed that omission of eIF1A slightly reduced initiation at AUG_108_ but promoted picking of near-cognate codons upstream, in particular UUG_41_ and to a lesser extent UUG_86_ (Figure 1D, lane 5). Therefore, whilst eIF1 is absolutely required for initiation, eIF1A is needed to maintain selection accuracy.

As flaviviral 5′ UTRs are known to be highly structured (21,45), we investigated the effect of RNA helicases on ZIKV translation initiation. Additional eIF4A, beyond that present in the eIF4F complex, was required for 48S complex assembly on capped ZIKV^5′^*^utr+^* RNA (Figure 1D, compare lanes 10 and 12). Similarly, omission of eIF4B, a cofactor that stimulates eIF4A activity and was recently reported to bind flaviviral genomes during infection (46), reduced the efficiency of translation initiation (Figure 1D, compare lanes 11 and 12). The eIF4A inhibitor hippuristanol (47) also abrogated 48S complex assembly in a dose-dependent manner (Supplementary Figure S1C), consistent with a recent study that showed ZIKV replication in A549 cells and primary human hepatocytes was inhibited by an alternative eIF4A inhibitor, silvestrol (48).

Since the ribosome-associated RNA helicase DHX29 has a described role in promoting initiation on highly structured RNAs (49), we investigated whether its inclusion could further enhance ZIKV translation initiation. However, addition of DHX29 led to a slight decrease in 48S complex assembly at the authentic AUG_108_ and UUG_41_ of capped ZIKV^5′^*^utr+^*RNA, in favour of other near-cognate initiation codons, UUG_86_ and CUG_102_, at low efficiency (Figure 1D, compare lanes 12 and 13). This is consistent with a previous report that DHX29 activity can influence upstream codon selection of the scanning complex (50).

### ZIKV replication requires genome circularization

**Figure 2.**
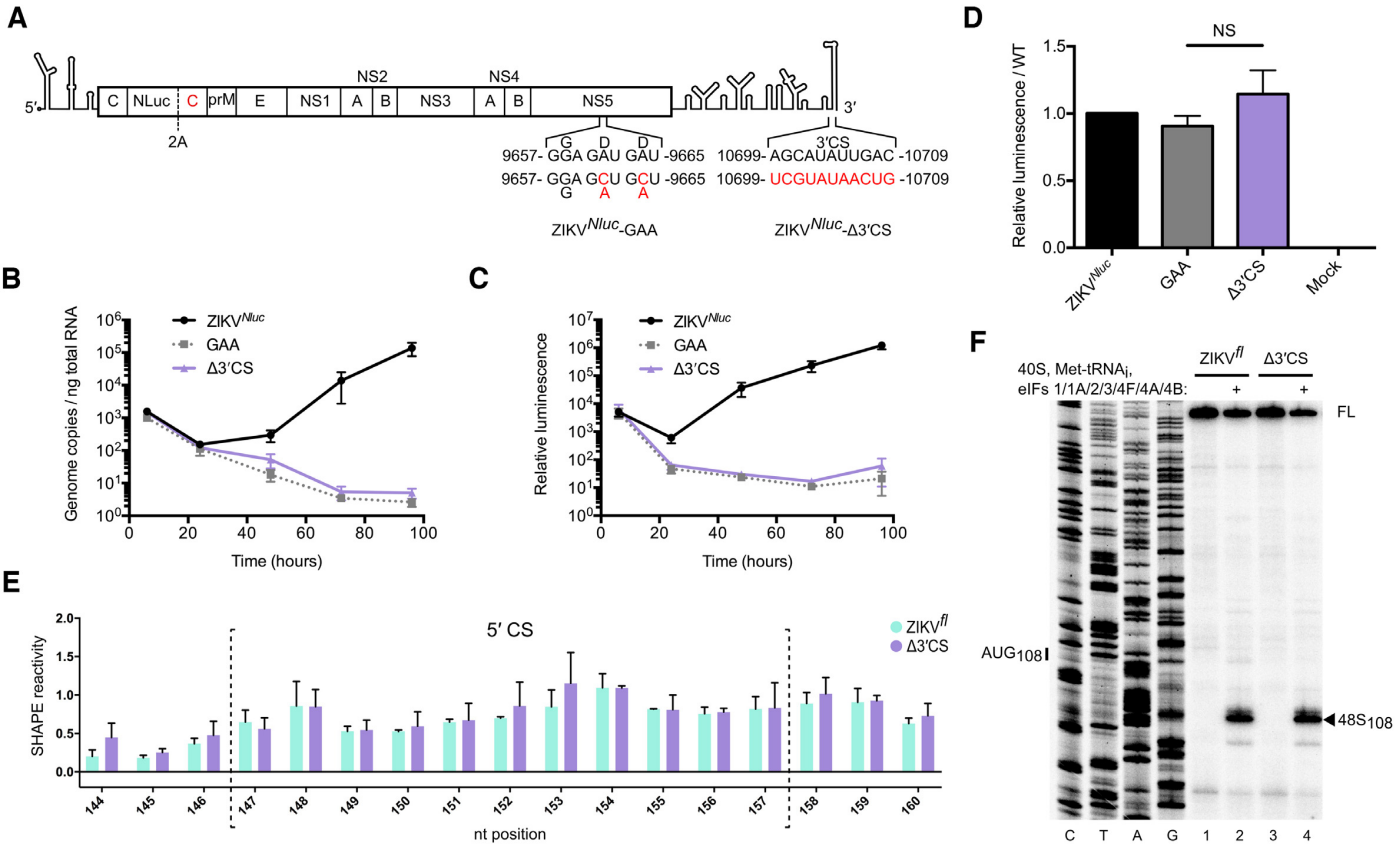
Genome circularization is required for ZIKV replication. (**A**) Schematic illustration of ZIKV*^Nluc^*. The first copy of the capsid gene is fused to a Nluc reporter and is separated from a second capsid sequence and the rest of the polyprotein by the FMDV 2A StopGo (40). ZIKV*^Nluc^*-GAA and ZIKV*^Nluc^*-Δ3′ CS polymerase mutations are indicated. (**B** and**C**) Time course of (B) ZIKV genome copy number quantified by qPCR and (C) luciferase activity normalized to total protein amount after electroporation of capped ZIKV*^Nluc^* RNA into Vero cells. Data are mean ± SEM for three independent experiments. (**D**) Luciferase activity normalized to total protein amount at 6 h post-electroporation relative to the WT control (full time course shown in C). Data are mean ± SEM of three independent experiments. ZIKV*^Nluc^*-GAA and ZIKV*^Nluc^*-Δ3′ CS mutants were compared by Mann–Whitney test (*P* = 0.40). NS, not significant. (**E**) SHAPE reactivity in the 5′ CS of ZIKV*^fl^* (green) and ZIKV*^fl^*-Δ3′ CS (purple). Nucleotides within the 5′ CS are enclosed by dotted brackets. Data are normalized SHAPE reactivities from three experiments, mean ± SD at each base. SHAPE reactivities in the 5′ CS region of ZIKV*^fl^* and ZIKV*^fl^*-Δ3′ CS mutant were compared by the Student's *t*-test and determined not to be significant. *P*-values are shown in Supplementary Table S2. (**F**) Toeprinting analysis of 48S complex assembly on capped ZIKV*^fl^* and ZIKV*^fl^*-Δ3′ CS RNA. Selected codons are labelled on the left and toeprints caused by 48S complex assembly are marked with a closed arrowhead on the right. FL, full-length.

It has been previously reported that DENV and WNV replication is dependent on intact 5′ and 3′ RNA–RNA interactions but that these are dispensable for translation (26,51,52). Therefore, we examined the impact of mutating one of these circularization elements, the 3′ CS, on ZIKV replication and translation in cells. We used a modified version of a plasmid-based reverse genetics system in which the ZIKV RNA (representative South American strain, BeH819015) possesses a nanoluciferase (Nluc) reporter fused to a duplicate copy of the viral capsid protein (shown schematically in Figure 2A) (40). Vero cells were electroporated with capped *in vitro* transcribed Nluc reporter ZIKV RNA (ZIKV*^Nluc^*) or a mutant RNA in which the 3′ CS was replaced with the 5′ CS to abrogate base pairing (ZIKV*^Nluc^*-Δ3′ CS; Figure 2A). Mutation of this region is known to be sufficient to disrupt DENV genome circularization (25,51). A replication negative control containing a G_664_DD → G_664_AA mutation in the viral polymerase active site was also examined (ZIKV*^Nluc^*-GAA; Figure 2A). Total RNA and cell lysates were harvested over 96 h and genome copy numbers were quantified by qPCR whilst luciferase was measured to monitor translation of the viral genome.

The genome copy number of all RNAs decreased between 6 and 24 h post-electroporation, consistent with loss of input RNA frequently observed in similar experiments (53). WT RNA recovered after 48 h (Figure 2B) but the ZIKV*^Nluc^*-GAA mutant could not replicate. ZIKV*^Nluc^*-Δ3′ CS mutant RNA showed similar replication kinetics to the defective polymerase mutant indicating that, similar to other flaviviruses, an intact 5′-3′ CS interaction is necessary for ZIKV replication. Translation of the viral genome followed the RNA replication kinetics (Figure 2C) and consistent with our *in vitro* reconstitution experiments was inhibited in the presence of the eIF4A inhibitor rocaglamide (Supplementary Figure S1D). Importantly, 6 h post-electroporation, at which time equivalent genome copy numbers of the three viruses were present, the amount of luciferase produced by ZIKV*^Nluc^*, ZIKV*^Nluc^*-GAA and ZIKV*^Nluc^*-Δ3′ CS was similar indicating that the WT and Δ3′ CS mutant RNAs are translated with the same efficiency in this experimental system (Figure 2D).

### Circularization inhibits *de novo* ZIKV translation initiation

A recent study has shown by RNA structure mapping that the majority of ZIKV genomic RNA in cells during infection is linear (32). This presents a limitation for examining the effect of genome circularization on translation in cell-based assays. To overcome this, we used the *in vitro* reconstitution technique to directly compare translation on linear and circular templates in a controlled system. We first *in vitro* transcribed and capped WT full-length ZIKV genomic RNA (ZIKV*^fl^*) lacking the capsid duplication or Nluc reporter (40). As this RNA contains the complete 5′ and 3′ UTRs we hypothesized that these could hybridize and provide a suitable template for evaluating the impact of circularization on translation initiation. To determine if this was the case we used SHAPE RNA structure analysis (54). In this technique, *in vitro* transcribed RNA is modified by incubation with *N*-methyl isatoic anhydride (NMIA) which reacts with the 2′-hydroxyl group of RNA. This modification is detected as a premature stop in a reverse transcription reaction, with unpaired bases characteristic of unstructured RNA being more reactive than those trapped in secondary structure elements.

Surprisingly, we observed that nucleotides in the 5′ CS had comparably high reactivity in ZIKV*^fl^* and ZIKV*^fl^*-Δ3′ CS, in which the 3′ CS was replaced with the 5′ CS as in the Nluc reporter RNA (Figure 2E), indicating that the CS is not hybridized in the ZIKV*^fl^* RNA *in vitro*. Consistently, the efficiency of 48S complex formation on capped ZIKV*^fl^* and ZIKV*^fl^*-Δ3′ CS RNAs was similar when examined using the *in vitro* reconstitution system followed by toeprinting analysis (Figure 2F). We found the same factor requirements for 48S complex assembly on the full-length ZIKV*^fl^* RNA as for the truncated ZIKV^5′^*^utr+^* RNA (Supplementary Figure S1E).

**Figure 3.**
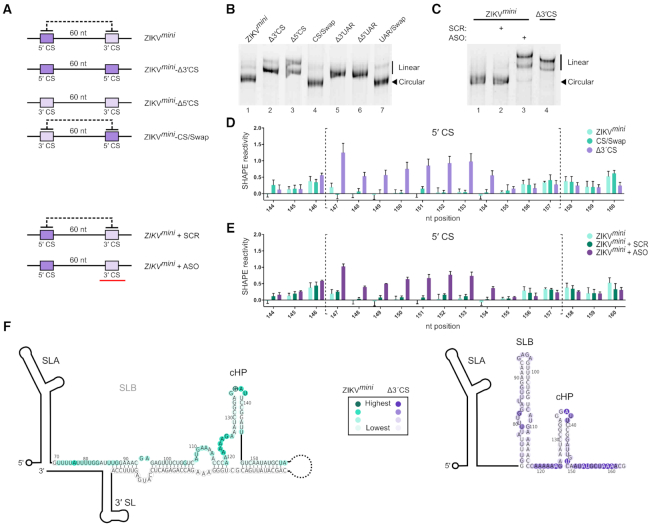
ZIKV*^mini^* RNA is circularized. (**A**) Schematic illustration of the ZIKV*^mini^* mutants examined. The dark and light purple represent the 5′ CS and 3′ CS, respectively. The black dotted line represents cyclization potential and the red bar represents the anti-sense oligo (ASO). (**B** and **C**) Native PAGE analysis of (B) ZIKV*^mini^* containing mutations in either the CS or UAR elements and (C) WT ZIKV*^mini^* after heating and snap-cooling in the presence or absence of ASO or scrambled oligo (SCR). (**D** and **E**) SHAPE reactivity in the 5′ CS of the WT and mutant ZIKV*^mini^* RNA. Nucleotides within the 5′ CS are enclosed by dotted brackets. WT SHAPE reactivity in light green is compared to (D) CS swap (dark green) and Δ3′ CS (purple) or to (E) WT annealed to SCR (dark green) or ASO (purple) RNA. Data are mean ± SD of normalized SHAPE reactivities at each base from three experiments. The same WT dataset is used in each panel as all conditions were examined simultaneously. SHAPE reactivities in the 5′ CS region of the WT and mutant minigenomes were compared by the Student's *t*-test. *P*-values are shown in Supplementary Table S2. (**F**) Heat map of SHAPE reactivities from a representative experiment overlaid onto ZIKV*^mini^* (left/green) and ZIKV*^mini^*-Δ3′ CS (right/purple) 5′ cyclization elements. The colour intensity represents increasing SHAPE reactivity as indicated in the box. See also Supplementary Figure S2.

As our SHAPE analysis showed that the *in vitro* transcribed ZIKV*^fl^* RNA adopts a linear conformation, we hypothesized that reducing the ∼10 kb separation of the UTRs may increase the likelihood of circularization. Therefore, we constructed a ZIKV minigenome (ZIKV*^mini^*) with a decreased linker length of 60 nt between the otherwise unaltered 5′ and 3′ UTRs. Similar constructs were previously used to dissect the role of circularization on flavivirus replication (29,31,55). We also made additional constructs in the ZIKV*^mini^* background that contained alterations within the cyclization elements at either the 5′ or 3′ ends (shown schematically in Figure 3A). Similar to the ZIKV*^fl^*-Δ3′ CS described above, to disrupt base pairing, the 3′ CS was replaced with the ZIKV 5′ CS to generate ZIKV*^mini^*-Δ3′ CS. Alternatively, the 5′ CS in the minigenome was replaced with the 3′ CS, also disrupting base pairing, to generate ZIKV*^mini^*-Δ5′ CS. In ZIKV*^mini^*-CS/Swap the 5′ and 3′ CS elements were swapped so as to restore base pairing and circularization potential. Corresponding mutations were also generated in the UAR regions of the minigenome to create ZIKV*^mini^*-Δ3′ UAR, ZIKV*^mini^*-Δ5′ UAR and ZIKV*^mini^*-UAR/Swap.

The conformation of the ZIKV minigenomes was first examined by native PAGE. Circularized RNAs should adopt a more compact structure and therefore migrate faster than those that are linear. Consistently, ZIKV*^mini^*-Δ3′ CS and ZIKV*^mini^*-Δ5′ CS migrated slower than the WT ZIKV*^mini^* (Figure 3B, compare lane 1 to 2 and 3). In contrast, ZIKV*^mini^*-CS/Swap migrated like WT (Figure 3B, compare lanes 1 and 4) indicating restoration of base pairing and circularization. Similar results were obtained for the UAR minigenome mutants (Figure 3B, compare lane 1 to 5, 6 and 7).

The structure of the ZIKV minigenomes was further analysed by SHAPE. The SHAPE profile in the 5′ region of the WT ZIKV*^mini^* had very low reactivity in the nucleotides of the 5′ CS, indicating that they are base paired (Figure 3D). In contrast, nucleotides corresponding to the 5′ CS in the ZIKV*^mini^*-Δ3′ CS were highly reactive, similar to that of the ZIKV*^fl^* RNA (compare Figure 3D to Figure 2E). Within the 5′ CS of the ZIKV*^mini^*-CS/Swap, nucleotides displayed reduced reactivity similar to that of WT ZIKV*^mini^*, indicating that swapping the 5′ and 3′ CS elements restores base pairing. Importantly, the SHAPE reactivity in the region surrounding the AUG_108_ start codon, the cHP stem and the 5′ CS element (Supplementary Figure S2) is consistent with recently described structures from multiple in-cell flaviviral RNA structural analyses (32,56,57). Combined with our native PAGE analysis, these results confirm that the WT and 5′-3′ CS swap ZIKV minigenomes are circularized whilst the Δ3′ CS ZIKV minigenome is linear.

**Figure 4.**
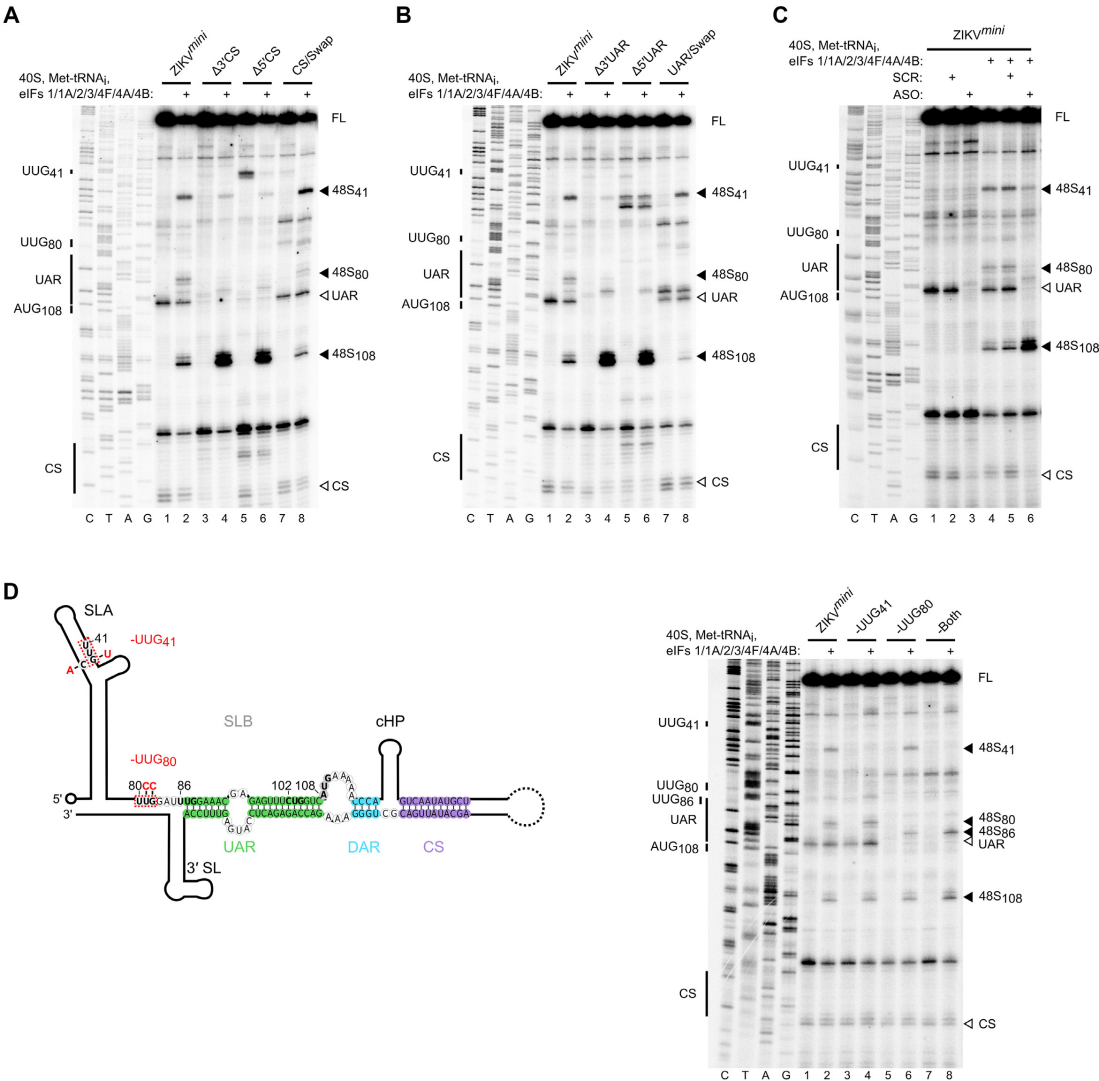
ZIKV genome circularization inhibits translation initiation. Toeprinting analysis of 48S complex assembly on WT capped ZIKV*^mini^* RNA and (**A**) ΔCS mutants, (**B**) ΔUAR mutants, (**C**) in the presence of scrambled (SCR) or anti-sense (ASO) RNA oligos and (**D**) upstream near-cognate codon mutants highlighted in red in the schematic on the left. Selected codons are labelled on the left of each gel and toeprints caused by 48S complex assembly are marked with a closed arrowhead on the right. Open arrowheads mark reverse transcriptase stops caused by hybridization of the 5′ and 3′ cyclization elements. FL, full-length. See also Supplementary Figure S3.

We next performed *in vitro* reconstitution on the capped ZIKV minigenome RNAs. In contrast to WT ZIKV*^fl^* RNA (Figure 2F), weak 48S complex assembly was detected at the initiating AUG_108_ on the WT ZIKV*^mini^* (Figure 4A, lane 2). Mutation of the 5′ or 3′ CS elements, abrogating circularization, increased 48S complex formation at this site (Figure 4A, compare lane 2 to lanes 4 and 6), whilst restoration of base pairing with the 5′-3′ CS swap mutation reduced 48S complex assembly to that of the WT ZIKV*^mini^* (Figure 4A, compare lanes 2 and 8). This indicates that the effect on translation initiation is not an artefact of the mutations introduced, but a direct effect of genome circularization. Similar to the CS mutations, mutation of the 5′ or 3′ UAR sequences also stimulated 48S complex formation (Figure 4B, compare lane 2 to lanes 4 and 6), with a 5′-3′ UAR swap substitution again reversing this effect (Figure 4B, compare lanes 2 and 8).

In order to ensure that differences other than circularization state between constructs were not responsible for these results, we examined the effect of adding a short antisense RNA oligo (ASO) complementary to the cyclization elements at the 3′ end of the WT ZIKV minigenome. Hybridization with the ASO, but not a scrambled oligo of equal length, retards the migration of the ZIKV*^mini^* in native PAGE in a manner similar to mutation of the 3′ CS (Figure 3C, compare lanes 2 and 3). The linear ASO-hybridized RNA is retarded to a slightly greater extent due to the mass of the annealed oligo. Linearization in the presence of the ASO, but not the scrambled oligo, was further confirmed by SHAPE analysis (Figure 3E). As shown in Figure 4C, the presence of the ASO rescues 48S complex formation at AUG_108_ on the WT ZIKV*^mini^* RNA whilst a scrambled RNA oligo does not (Figure 4C). Our results therefore show that the circular form of the ZIKV RNA is not permissive for *de novo* translation initiation.

### Genome circularization impairs scanning

Interestingly, whilst ZIKV RNA circularization reduced 48S complex assembly at AUG_108_, complex assembly at two upstream near-cognate codons (UUG_41_ and UUG_80_) was enhanced (Figure 4A and B, compare lanes 2 and 8 with lanes 4 and 6). The stimulation of 48S complex assembly at sites upstream of AUG_108_ on the circularized RNA occurred despite the presence of an excess of eIF1A, which was shown to regulate selection of upstream codons in the linear form (Figure 1D). This is consistent with circularization of the ZIKV RNA inducing a scanning defect, potentially caused by the inability of the scanning 43S complex to penetrate the RNA secondary structure around AUG_108_ in the circular form. Addition of increasing amounts of eIF4F or DHX29 was not sufficient to overcome the inhibition of scanning imposed by circularization (Supplementary Figure S3).

We next examined the impact of mutating UUG_41_ and/or UUG_80_ within the context of the circularized WT ZIKV*^mini^*. The mutations introduced are shown in Figure 4D. UUG_80_ was mutated to UCC, whilst UUG_41_ was replaced by UUU with a compensatory C21A mutation to maintain base pairing in SLA. The introduced mutations abrogated 48S complex assembly at UUG_41_ and UUG_80_ (Figure 4D, compare lane 2 to lanes 4 and 6). Interestingly, simultaneous mutation of both UUG_41_ and UUG_80_ did not enhance 48S complex assembly at AUG_108_, but instead UUG_86_ at the 5′ leading edge of the UAR was selected (Figure 4D, lane 8). These data suggest that initiation at UUG_41_ or UUG_80_ is not strictly required for the inhibition of 48S assembly seen at AUG_108_ in the circular conformation but that the RNA structure alone is sufficient to mediate this effect.

### Genome circularization also inhibits *de novo* translation initiation in DENV

**Figure 5.**
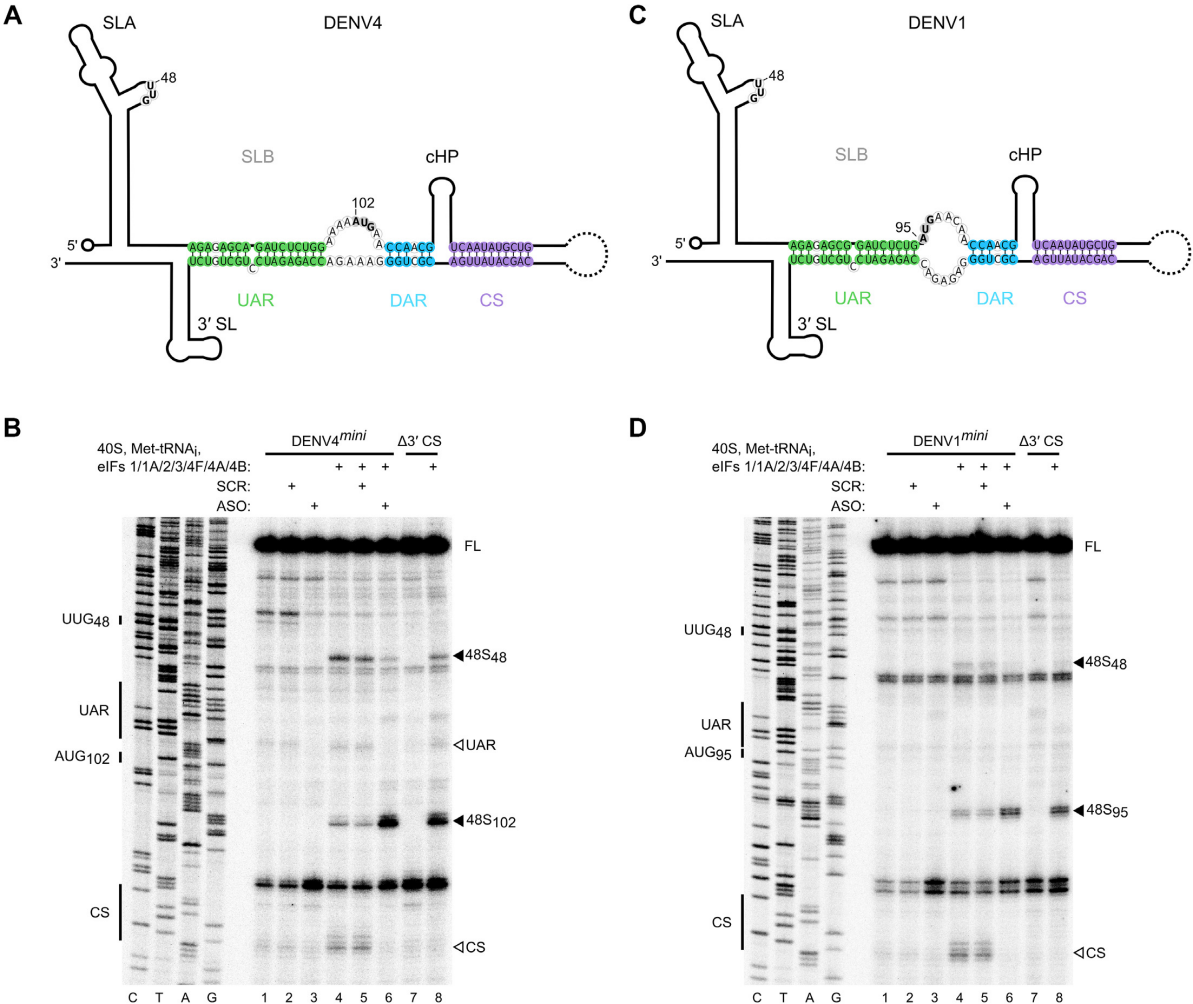
Circularization inhibits translation initiation on DENV. (**A** and **C**) Schematics of the (A) DENV4 and (C) DENV1 genomes in the circular conformation. Nucleotides of the UAR, DAR and CS are shown in green, blue and purple, respectively. The initiating AUG and upstream near-cognate codons discussed in the text are in bold. (**B** and **D**) Toeprinting analysis of 48S complex assembly on capped (B) WT and Δ3′ CS DENV4*^mini^* or (D) WT and Δ3′ CS DENV1*^mini^* RNAs. Selected codons are labelled on the left and toeprints caused by 48S complex assembly are marked with a closed arrowhead on the right. Open arrowheads mark reverse transcriptase stops caused by hybridization of the 5′ and 3′ cyclization elements. Scrambled (SCR) or anti-sense (ASO) RNA oligos were annealed to DENV1/4*^mini^* RNAs prior to the assembly reaction as indicated. FL, full-length.

Since it is a common feature of all flaviviruses, we investigated if circularization also affects translation initiation of DENV. We examined 48S complex assembly on minigenome RNAs that contained the 5′ and 3′ UTRs of either DENV1 or DENV4, the UTRs of which are the most divergent between the DENV serotypes (Figure 5A and C). We used a similar approach to that used for the analysis of the ZIKV*^mini^* RNA by mutating the 3′ CS or adding a short ASO complementary to the 3′ cyclization elements to disrupt genome circularization. As in ZIKV, circularization inhibited *de novo* translation initiation upon DENV1 and DENV4 minigenome RNAs, whilst disruption of circularization enhanced 48S complex formation on the authentic initiating AUG (AUG_95_ in DENV1 and AUG_102_ in DENV4; Figure 5B and D, compare lane 4 to lanes 6 and 8). Like ZIKV, enhanced picking of the upstream UUG_48_ was observed for DENV RNAs, consistent with a scanning defect in the circulariszed form. Overall, our data indicates that genome circularization is a conserved mechanism employed by different flaviviruses to control genome usage.

## DISCUSSION

To efficiently replicate, single-stranded positive-sense RNA viruses face a conundrum. As with most viruses, they are dependent on the cellular translation machinery to make viral proteins required for replication and have evolved intricate mechanisms to compete with host mRNAs for eIFs and ribosomes. However, for infection to progress they must decrease translation of their genome in order to free it as a template for negative strand synthesis, since only after this event can new positive-sense genomes be mass-produced for packaging into progeny virions. Here, we have demonstrated that mosquito-borne flaviviruses can inhibit *de novo* translation initiation through genome circularization.

A requirement for viral genome circularization via direct RNA–RNA interactions is a hallmark of flaviviral replication (25,28,51,52,58). Previously inferred from sequence comparison and mutagenic analyses, the emergence of high-throughput sequencing techniques has enabled these interactions to be detected in cells during infection using proximity cross-linking of RNA elements followed by deep sequencing, as recently reported (56). Although the authors observed circularization during infection, information on the relative amount of circular versus linear RNA present in the cell was lacking. Our initial efforts to examine the impact of 5′-3′ interaction on translation of the full-length viral RNA were not successful as we found that, like ZIKV*^fl^*-Δ3′ CS mutant RNA, *in vitro* transcribed WT ZIKV*^fl^* was not circularized. Using in-cell SHAPE RNA structure probing, Li *et al.* (32) demonstrated that at a late time point post-infection (72 h), ZIKV RNAs are also mainly linear, complicating analysis of the effect of genome circularization on translation. We overcame this problem by performing *in vitro* reconstitution experiments on ZIKV minigenome RNAs, allowing us to reveal the inhibitory effect of circularization on translation initiation (Figure 4). Importantly, the structure of our minigenome RNAs matches that recently described as occurring in full-length viral RNA during infection by three independent groups (32,56,57), demonstrating the relevance of our template RNAs.

**Figure 6.**
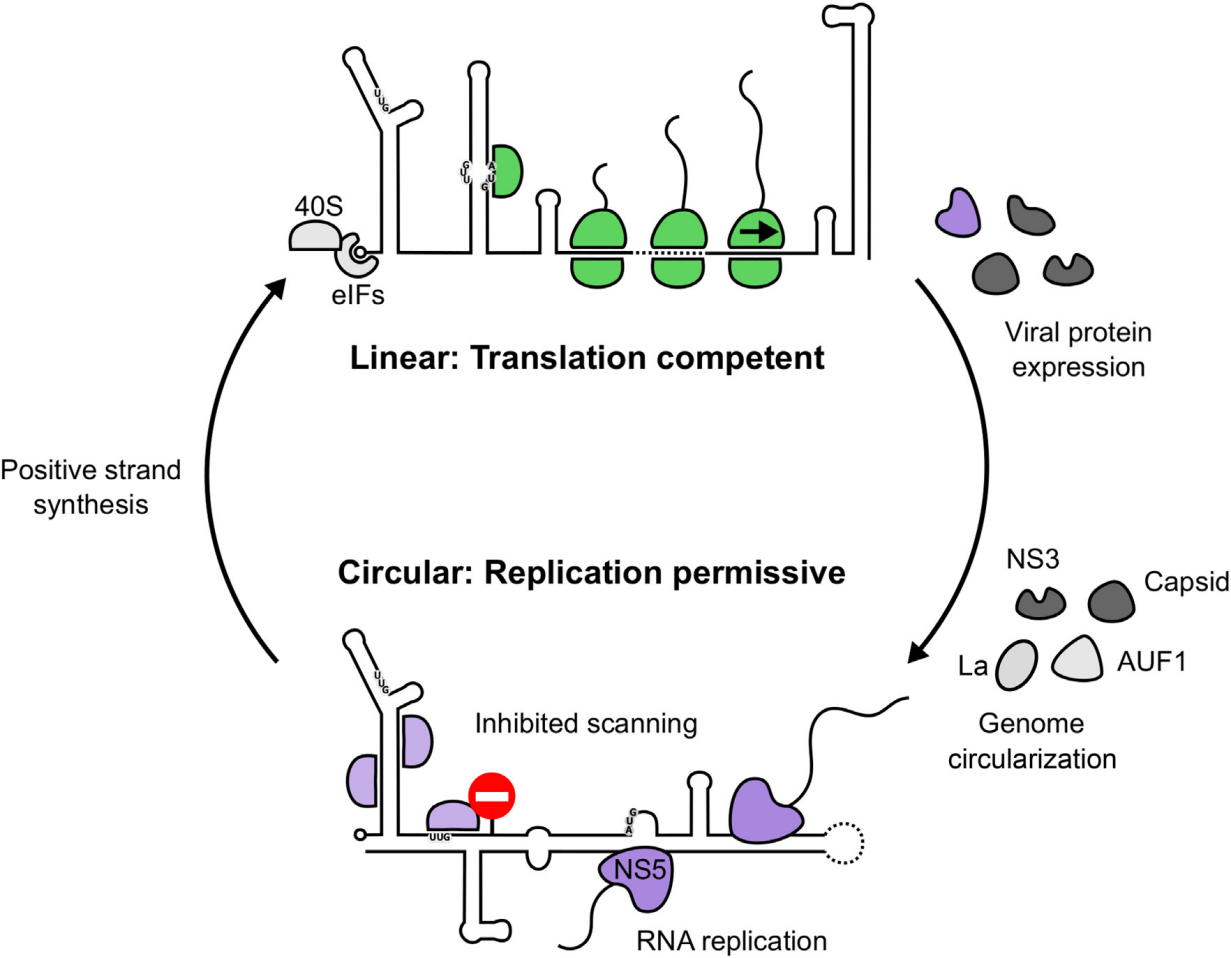
Model for the control of translation initiation by genome circularization. Linear genomic RNA recruits eIFs and 40S ribosomal subunits (pale grey) in a cap-dependent manner. Secondary structure in the 5′ UTR is effectively resolved by helicase eIF4A to allow translation initiation from the authentic initiation codon, indicated by a green 48S complex. Following genome circularization, promoted by viral and host factors (64–68) and likely occurring at the ER membrane, scanning ribosomes are impeded by extensive RNA structure formed by hybridization of 5′ and 3′ cyclization elements. Decreased translation from the authentic AUG permits efficient genome replication by NS5^pol^ (purple).

The mechanism by which viral genome circularization inhibits *de novo* translation initiation is likely through inhibition of scanning (see model in Figure 6). We observed increased 48S assembly on near-cognate codons upstream of the 5′ end of the UAR in the circular minigenome RNAs. We found that mutating upstream UUG codons did not enhance 48S assembly at the authentic AUG (Figure 4D), suggesting that the scanning 43S complex cannot penetrate the RNA structures found exclusively in the circularized form. Addition of the DExH-box RNA helicase DHX29, which enhances 48S complex formation on highly structured RNAs (49), was not sufficient to overcome this block in scanning (Supplementary Figure S3). DHX29 has been shown to promote translation on RNA containing a stable stem in the 5′ UTR with a predicted free energy of −27.6 kcal/mol (49), similar to that of the ZIKV UAR in the circular conformation (ΔG = −26.7 kcal/mol), as calculated in Mfold (59). Therefore, the reason for this inability of DHX29 to promote 48S complex assembly on circularized ZIKV RNA is not immediately clear. However, regions of flavivirus genomic RNA are known to fold into complex tertiary structures important for replication, subgenomic RNA production and host adaptation (60–62). It is possible that in the circularized form, the 5′-3′ duplex adopts a tertiary conformation that cannot be resolved by DHX29.

During infection, flaviviral genomic RNA is recruited to the ER (12) but the RNA conformation upon recruitment is unknown. Liu *et al.* (31) reported that the viral NS5^pol^ binds more efficiently to SLA in the linear form of DENV4 RNA *in vitro*. As the viral polymerase, NS5^pol^ forms a key part of the viral replication complex and is tethered to the ER membrane through its interaction with the viral NS3 helicase (NS3^hel^) and its membrane-associated cofactor NS2B (63). DENV NS3^hel^ (64) and the WNV capsid protein (65) have been reported to impact viral genome structure. Similarly, cellular proteins AUF1 isoform p45 (66,67) and La (68) were shown to bind the 5′ and 3′ termini to bring them into close proximity, promoting viral replication. However, the relative contributions and importance of each of these viral and cellular factors in promoting or stabilizing viral genome 5′-3′ hybridization remains to be clarified. Other cellular or viral factors may also negatively influence genome circularization during infection to promote efficient translation of viral proteins. Once identified it will be of interest to examine the effect of such factors in the *in vitro* system presented here. Identifying and targeting these factors which influence flavivirus genome circularization, to disrupt the delicate balance between translation and replication, potentially opens up avenues for developing novel anti-viral therapeutics.

Flavivirus infection induces cellular membrane reorganization into vesicles that provide sites for viral genome replication (69), from which newly synthesized viral RNAs may be extruded for further translation or packaging. Although improvements in cellular imaging techniques have facilitated a more detailed description of these virus-induced vesicles (70), experimental evidence of early events in their assembly and the organization of viral proteins and RNA within them is still lacking. It is also currently unknown how the accessibility of the viral RNA to host translation machinery is affected by its sequestration at these replication sites.

Here, translation initiation has been fully reconstituted *in vitro* on a member of the *Flavivirus* genus. We found that a 5′ cap and all the canonical cap-dependent eIFs were required for translation initiation on ZIKV RNA (Figure 1C and D), whereas only a small subset of the same purified factors were sufficient for initiation on the CSFV IRES. Consistently, we and others have demonstrated that antiviral cap-binding proteins are capable of efficiently inhibiting flaviviral translation and replication *in vitro* and *in vivo* (71,72). Interestingly, host cell translation shutoff has been shown to occur upon infection with different flaviviruses but, remarkably, ZIKV and DENV RNAs are resistant to this inhibition (73), consistent with reports that both viruses may also translate their genomes in a cap-independent manner by an unknown mechanism (74,75). Although our *in vitro* reconstitution experiments demonstrate a requirement for the canonical translation factors for efficient ZIKV cap-dependent translation, it remains to be determined if flavivirus RNAs can interact with these factors in a non-canonical fashion.

In this report we have revealed an unusual mechanism of translational control used by flaviviruses. Our findings fill a major hole in the understanding of the life cycle of this important class of human pathogens and extends the multiple roles performed by dynamic viral RNA structures at different stages of infection.

## Supplementary Material

gkz686_Supplemental_File
