# Supplementary material for: Circularization of flavivirus genomic RNA inhibits de novo translation initiation
Source: Nucleic Acids Res. 2019 Aug 8;47(18):9789–802. doi: 10.1093/nar/gkz686 (PMC6765113; doi:10.1093/nar/gkz686)
Supplement: gkz686_Supplemental_File [file gkz686_supplemental_file.pdf]

Supplementary Information for

Circularisation of flavivirus genomic RNA inhibits *de novo* translation initiation.

Thomas J. Sanford[1], Harriet V. Mears[1], Teodoro Fajardo Jr[1], Nicolas Locker[2], Trevor R. Sweeney[1]*.

1 Division of Virology, Department of Pathology, University of Cambridge, Addenbrooke's Hospital, Hills Road, Cambridge, UK.
2 Faculty of Health and Medical Sciences, School of Biosciences and Medicine, University of Surrey, Guildford, UK.

*Correspondence to Trevor Sweeney
Address: Division of Virology, Department of Pathology, University of Cambridge, Box 237 Level 5 Lab Block, Addenbrooke's Hospital, Hills Road, Cambridge, CB2 0QQ, UK.
Email: ts629@cam.ac.uk
Phone: +44(0)1223336072

This PDF file includes:

Figures. S1 to S3
Table S1 and S2

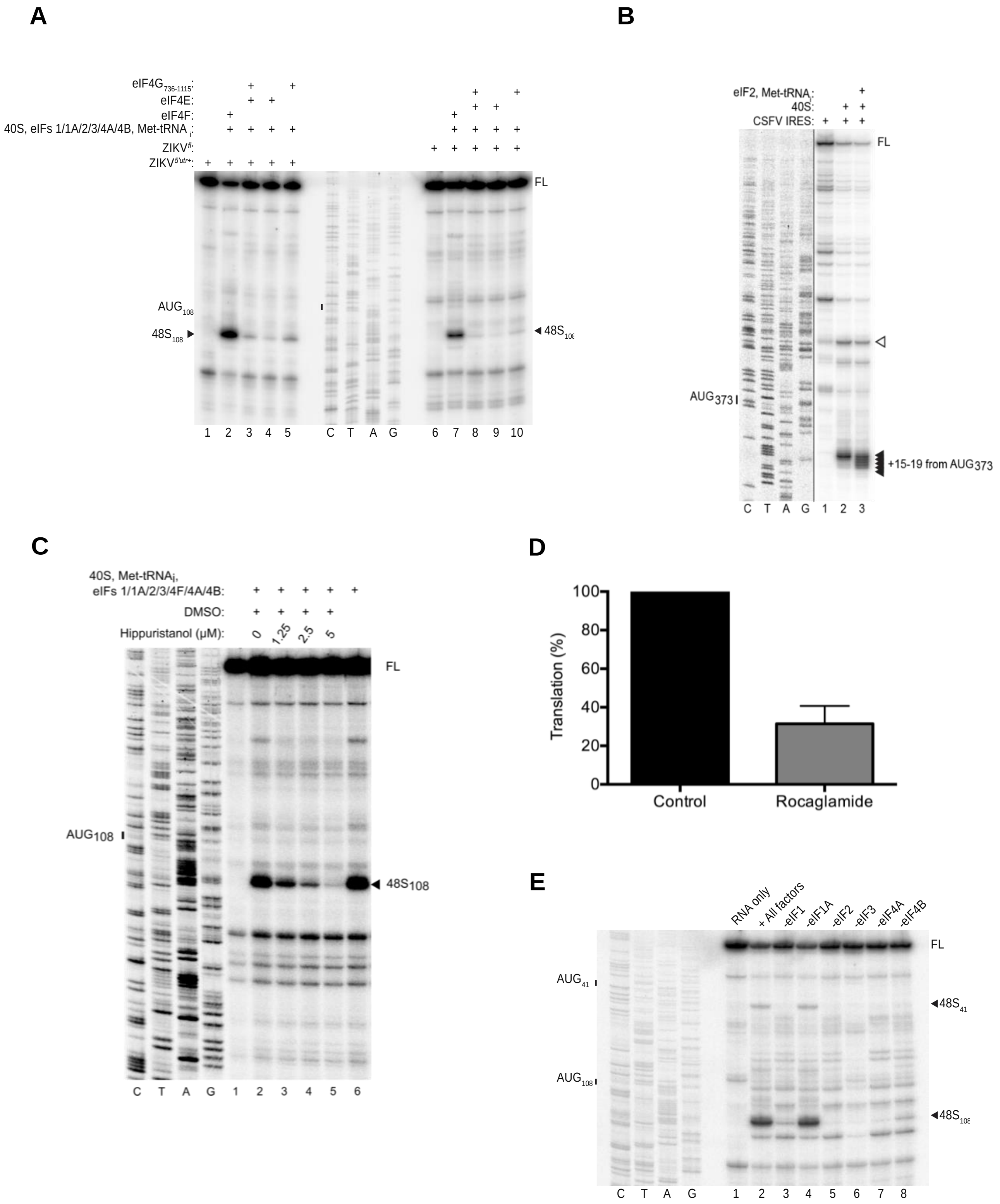
A
eIF4G736-1115:
eIF4E:
eIF4F:
40S, eIFs 1/1A/2/3/4A/4B, Met-tRNAi:
ZIKVfl:
ZIKV5'utr+:
FL
AUG108
48S108
1 2 3 4 5 C T A G 6 7 8 9 10
B
eIF2, Met-tRNAi:
40S:
CSFV IRES:
FL
AUG373
+15-19 from AUG373
C T A G 1 2 3
C
40S, Met-tRNAi,
eIFs 1/1A/2/3/4F/4A/4B:
DMSO:
Hippuristanol (μM):
0
1.25
2.5
5
FL
AUG108
48S108
C T A G 1 2 3 4 5 6
D
Translation (%)
100
80
60
40
20
0
Control
Rocaglamide
E
RNA only
+ All factors
-eIF1
-eIF1A
-eIF2
-eIF3
-eIF4A
-eIF4B
FL
AUG41
48S41
AUG108
48S108
C T A G 1 2 3 4 5 6 7 8

Figure S1. Translation factor requirements on different viral RNAs

A) Toeprinting analysis of 48S complex formation upon capped ZIKV$^{5'utr+}$ and ZIKV^{fl} RNA. AUG_{108} is labelled on the left and marked with a bar in the gel. Toeprints caused by 48S complex assembly are marked with a closed arrowhead. FL, full length cDNA signal.

B) Toeprinting analysis of 48S complex formation upon CSFV IRES. Toeprints caused by 48S complex assembly are marked with a closed arrowhead on the right. RT stops caused by IRES/40S interactions are marked with an open arrowhead on the right. FL, full length. The black line demarks different exposures of the same gel.

C) Toeprinting analysis of 48S complex formation upon capped ZIKV$^{5'utr+}$ at the indicated concentrations of hippuristanol. Selected codons are labelled on the left and toeprints caused by 48S complex assembly are marked with a closed arrowhead on the right. Lanes with 5% DMSO are indicated. FL, full length.

D) Luciferase activity at 6 hrs post electroporation of capped ZIKV^{Nluc} RNA in the presence of 30 nM rocaglamide A relative to the absence of drug. Cells were pre-treated with rocaglamide A for 3 hours before electroporation. Rocaglamide was maintained during infection. Data are mean+/-SEM of three independent experiments.

E) Toeprinting analysis of 48S complex formation upon capped ZIKV^{fl} RNA in the presence or absence of specific eIFs. Selected codons are labelled on the left. Toeprints caused by 48S complex assembly are marked with a closed arrowhead on the right. FL, full length cDNA signal.

Related to Figure 1.

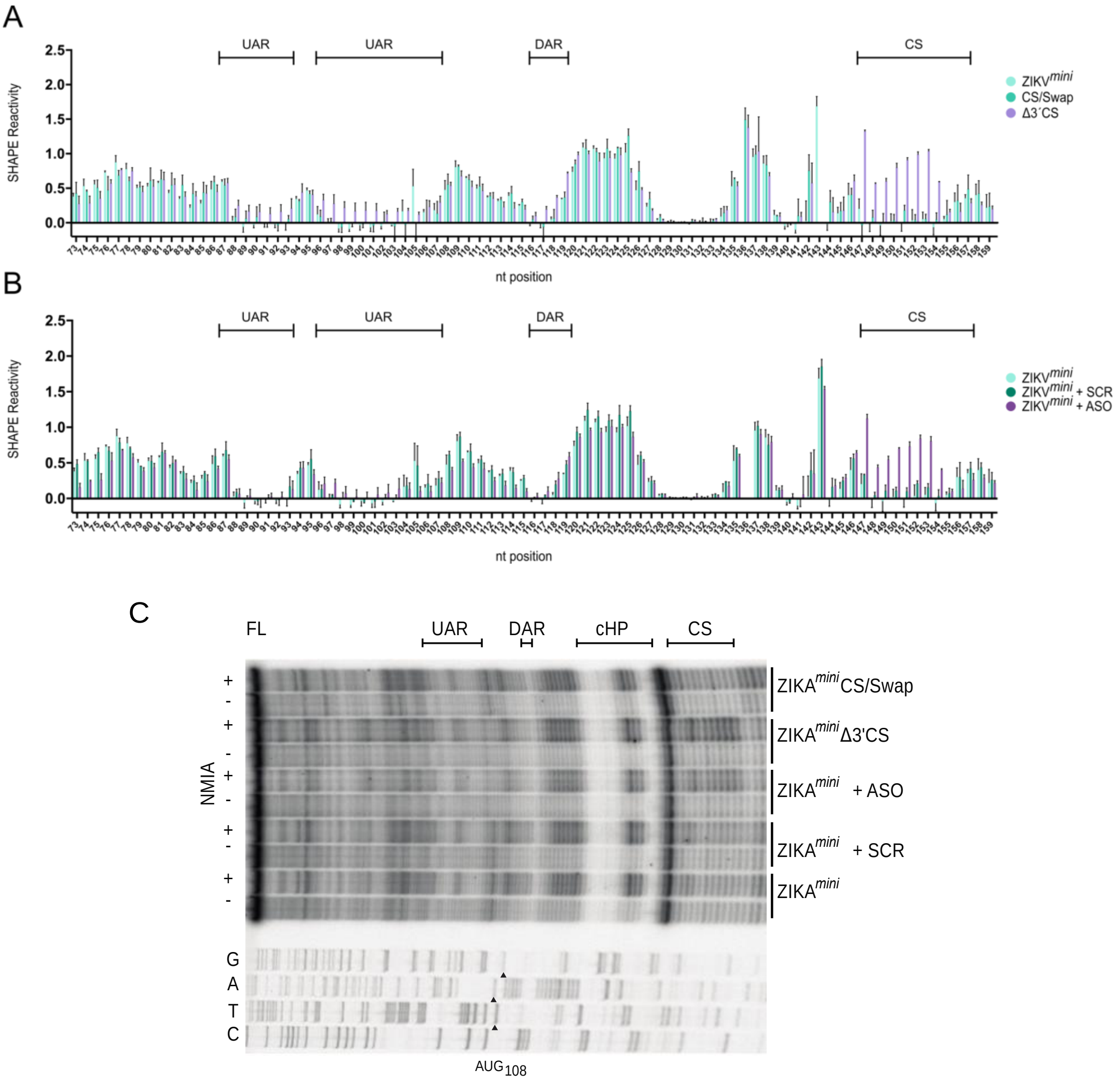

Figure S2. SHAPE analysis of the ZIKV^{mini} RNAs

A, **B**) Wildtype (WT) ZIKV^{mini} RNA SHAPE reactivity in light green is compared to (A) CS swap (dark green) and Δ3′ CS (purple) or to (B) WT annealed to a scrambled oligo (SCR) (dark green) or anti-sense oligo (ASO) (purple) RNA. SHAPE reactivities were calculated relative to the top 10% of reactivities from nt 73-159. Data are mean+/-SD of normalised SHAPE reactivities at each base from three experiments. The same wildtype dataset is used in each panel as all conditions were examined simultaneously.

C) Representative gel used to calculate SHAPE reactivities.

Related to Figure 3.

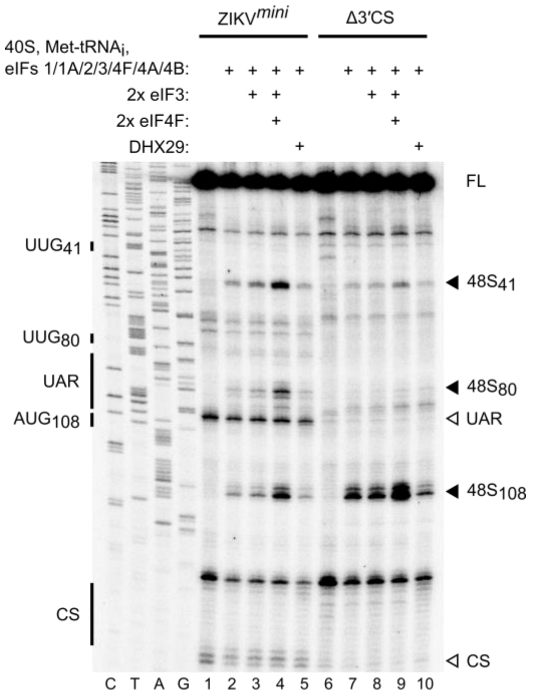

Figure S3. Addition of more eIF4F or DHX29 does not overcome the circularisation induced scanning defect

Toeprinting analysis of 48S complex assembly on capped ZIKV^{mini} or ZIKV^{mini}-Δ3′ CS using the conditions illustrated. Selected codons are labelled on the left and toeprints caused by 48S complex assembly are marked with a closed arrowhead on the right. Open arrowheads mark RT stops caused by hybridization of the 5′ and 3′ cyclisation elements. FL, full length. Related to Figure 4.

ZIKV ASO	AGCATATTGACGCTGGGAAAGACCAGAGACTC**CTATAGTGAGTCGTATTA**
DENV1 ASO	CAGCATATTGACGCTGGGAGAGACCAGAGATCCTGCTGTCT**CTATAGTGAGTCGTATTA**
DENV4 ASO	CAGCATATTGACGCTGGGAAAGACCAGAGATCCTGCTGTCT**CTATAGTGAGTCGTATTA**
SCR	ATGGCAAACCCAGATTGCTATTCCAAACGTCT**CTATAGTGAGTCGTATTA**

Table S1. Primers
DNA used as a template for transcription of RNA oligos. The T7 promoter is shown in bold.

nt	ZIKV^{fl} vs ZIKV^{fl}-Δ3′ CS	ZIKV^{mini} vs ZIKV^{mini} CS/Swap	ZIKV^{mini} vs ZIKV^{mini} Δ3′ CS	ZIKV^{mini} vs ZIKV^{mini}+ASO	ZIKV^{mini} vs ZIKV^{mini}+SCR
144	0.107	0.042	0.187	0.033	0.092
145	0.100	0.836	0.928	0.049	0.318
146	0.388	0.761	0.122	0.072	0.572
147	0.508	0.089	0.004	0.001	0.444
148	0.975	0.335	0.004	0.002	0.422
149	0.845	0.629	0.006	0.002	0.211
150	0.572	0.918	0.005	0.001	0.361
151	0.845	0.090	0.003	0.002	0.278
152	0.422	0.998	0.003	0.002	0.189
153	0.315	0.935	0.003	0.001	0.320
154	0.980	0.220	0.003	0.002	0.244
155	0.940	0.715	0.063	0.134	0.948
156	0.712	0.858	0.287	0.127	0.560
157	0.948	0.487	0.450	0.068	0.603
158	0.444	0.884	0.239	0.205	0.734
159	0.869	0.974	0.904	0.944	0.977
160	0.371	0.432	0.054	0.089	0.209

Table S2. Statistical significance of differences between SHAPE reactivities of nucleotides (nt) in the 5′ CS element.

Differences in mean SHAPE reactivities from three independent experiments were compared using an unpaired two-tailed Students t-test. P values calculated in GraphPad Prism and rounded to four decimal places are shown. P values <0.05 (highlighted in green) were deemed statistically significant.

Related to Figures 2 and 3.
